# Editorial: Special Issue Development of Micro and Nano Systems for the Drug Delivery

**DOI:** 10.3390/pharmaceutics14071440

**Published:** 2022-07-09

**Authors:** Giovanna Della Porta

**Affiliations:** 1 Department of Medicine, Surgery and Dentistry “Scuola Medica Salernitana”, University of Salerno, 84081 Baronissi, SA, Italy; gdellaporta@unisa.it; Tel./Fax: +39-089965234; 2 Interdepartmental Research Center for Biomaterials, University of Salerno, 84084 Fisciano, SA, Italy

In this Issue, I have collected ten research papers and four review articles trying to describe the technologies that have evolved in the past ten years for the development of micro and nano systems for drug carry, targeting and delivery. The review papers included research scenarios of nano-emulsions formulations for nose-to-brain delivery, taking into account that the blood–brain barrier (BBB) is one of the major issues and limiting the drugs targeting the brain [1]; the bio-inspired and in situ self-assembly of polypeptides recently proposed for potential challenges in clinical treatment [2]; nanoparticles inducing ferroptosis for tumor targeting and immunomodulation [3]; and different nanomedicine approaches to clinical malaria [4] in order to better explain how nanoscience can contribute to achieve solutions. On the other hand, for an accurate device formulation and engineering, its size tailoring and drug encapsulation efficiency are, in several cases, still a technological challenge for the near future. In this sense, smart nano-materials and related innovative processes and technologies for their fabrication can be extremely promising for both pharmaceutical and biomedical fields.

Research articles were selected to describe advanced nano-formulations for drug delivery and related fabrication technologies, such as high-energy microfluidization technique to obtain nano-emulsion with vesicles size of 200–250 nm [5], procedures to organize pH-sensitive thin films [6], protocols to obtain PEGylated liposomes [6], and superparamagnetic iron oxide nanoparticles (SPIONs) [7]. All these formulations have been reported to successfully encapsulate anticancer drugs for a more precise and better targeted delivery.

From nanovesicles and nanosomes up to micro/nano advanced materials, the papers selected explored the different solutions adopted and the several approaches attempted to formulate complex micro/nano devices for active principle storage and delivery versus specific target. Advanced formulations including iron oxide mesoporous magnetic nanostructures (IO-MMNs) fabricated via new chemical synthesis [8], bio-inspired cell-derived nano-vesicles (CDNs) with unique properties in terms of binding and target cell uptake by and intrinsic biological activities [9], and nanostructured Lipid Carriers dispersions stabilized with a carbohydrate cryoprotectants [10], are all examples of innovative and nanostructures materials developed for advanced formulations.

Finally, it is my opinion that the development of robust and reproducible in-vitro models is extremely important for the drug formulation pre-screening and testing. However, monolayer 2D culture are not proper usefull for the scope, whereas, 3D culture coupled with advanced bioreactor for dynamic input delivery are fundamental instruments for exploring the effectiveness of those innovative formulations. As an example, was included in the collection a study on formulation properties acquired under physiological flow using a modified plate-flow chamber. By adopting fluorescently labeled human platelets and endothelial progenitor cells (EPCs) it was possible to monitor cells response in real-time using fluorescent imaging [11]. 

All papers selected covered issues to explore how the research field of drug delivery and nanomedicine is evolving and what the future might bring.
